# Autophagy inhibition improves sensitivity to the multi-kinase inhibitor regorafenib in preclinical mouse colon tumoroids

**DOI:** 10.3389/fcell.2025.1631116

**Published:** 2025-07-23

**Authors:** Giulia Agostini, Morgane Leprovots, Jérôme Jeandriens, Anne Lefort, Frédérick Libert, Francesco Sclafani, Ingrid Langer, Alain Hendlisz, Marie-Isabelle Garcia

**Affiliations:** ^1^IRIBHM, Jacques E. Dumont, Faculty of Medicine, Université Libre de Bruxelles ULB, Brussels, Belgium; ^2^Institut Jules Bordet, Hôpital Universitaire de Bruxelles (HUB), Université libre de Bruxelles (ULB), Brussels, Belgium; ^3^BRIGHTcore ULB-VUB and Institute of Interdisciplinary Research in Human and Molecular Biology (IRIBHM), Université Libre de Bruxelles, Brussels, Belgium

**Keywords:** colorectal cancer, regorafenib, chemoresistance, EMT, autophagy, organoids, tumoroids, fibroblasts

## Abstract

Colorectal cancer (CRC) remains the second leading cause of cancer-related deaths worldwide, with its incidence continuing to rise. Regorafenib, a multi-kinase inhibitor approved for palliative treatment, has been shown to extend survival in patients with metastatic CRC (mCRC) who have failed standard therapies. However, its clinical benefit is limited to a subset of patients, is typically short-lived, and is often accompanied by significant toxicity. The mechanisms by which CRC cells develop resistance to regorafenib remain incompletely understood. In this study, we investigated the mechanisms of regorafenib resistance using a preclinical mouse colon organoid model. Transcriptomic analysis of Apc wild-type and Apc-deficient organoids treated with regorafenib revealed upregulation of epithelial-to-mesenchymal transition (EMT), alterations in the secretome, and increased activation of phosphorylated Erk1/2. Notably, co-treatment with an autophagy inhibitor suppressed regorafenib-induced EMT and its associated secretory phenotype, leading to reduced cell proliferation and enhanced apoptosis in mouse organoids. The efficacy of this drug combination was further supported by cell viability assays in human CRC cell lines. In contrast, primary mouse colon fibroblasts exhibited greater resistance to both single-agent and combination regorafenib treatments. In summary, our findings using an organoid model suggest that autophagy inhibition may represent a promising strategy to overcome chemoresistance to regorafenib in mCRC patients.

## 1 Introduction

Colorectal cancer (CRC) is the second most frequently diagnosed cancer in men (after prostate and lung cancers) and the second most common in women (after breast cancer). According to World Health Organization statistics, 519,820 people in Europe were diagnosed with CRC in 2022, accounting for 12.7% of all new cancer cases (Globocan, 2022). By 2040, the global burden of CRC is projected to reach 3.2 million new cases and 1.6 million deaths annually, with the majority occurring in countries with high or very high Human Development Index (Morgan et al., 2023). CRC develops through a well-characterized process known as the adenoma-carcinoma sequence, which begins with the transformation of normal colonic epithelium into benign adenomatous polyps. Over time, these polyps may progress to invasive adenocarcinoma (Nguyen et al., 2020). Regular screening is the most effective strategy for early detection. In individuals with polyps, it serves as secondary prevention, while in healthy individuals, it functions as primary prevention (Li et al., 2024). Standard treatments for localized CRC include surgery, often combined with chemotherapy and/or radiotherapy (Brenner et al., 2014). Despite surgical resection, some patients experience disease recurrence, and others present with metastatic disease at diagnosis (Guraya, 2019). For these patients, therapeutic options remain limited.

Regorafenib is a multi-kinase inhibitor approved for use in patients with chemo-refractory metastatic CRC (mCRC) (de la Fouchardiere, 2018). It targets key pathways involved in CRC progression including angiogenesis via inhibition of VEGFR1, VEGFR2, VEGFR3, TIE2, PDGFR, FGFR1, and FGFR2; proliferation via inhibition of c-KIT, RAF1, BRAF, and RET; and metastasis via inhibition of VEGFR2, VEGFR3, and PDGFR (Arai et al., 2019). However, its clinical efficacy is often limited by the rapid development of drug resistance. Intrinsic resistance has been linked to constitutive activation of the RAF/MAPK/ERK pathway in tumors harboring mutations in *KRAS*, *BRAF* or *PI3KCA* (Goetz et al., 2014). Acquired resistance mechanisms include induction of epithelial-mesenchymal transition (EMT) and cellular senescence (Kehagias et al., 2022). Bulk RNA sequencing of patient-derived xenografts has associated resistance with elevated basal *EPHA2* expression (Lafferty et al., 2021). More recently, Mao et al. (2024) defined a gene expression signature in patient-derived tumor organoids in response to regorafenib, while transcriptomic analysis of tissues from treatment-sensitive and -resistant patients revealed involvement of metabolic pathways and P53 and ERBB signaling (Liu et al., 2025). Despite these advances, the mechanisms underlying regorafenib resistance and its toxicity to healthy tissues remain incompletely understood. A deeper understanding of these processes is essential to improve patient outcomes.

Recent breakthroughs in stem cell biology have identified key signaling pathways regulating stem cell self-renewal and differentiation in the adult colon (Barker et al., 2007; Sato et al., 2011). These findings have enabled the development of organoid technology, which involves culturing self-organizing stem cells in extracellular matrix scaffolds to form structures that mimic native tissue architecture (Qu et al., 2024). These three-dimensional *in vitro* systems closely replicate the cellular composition, behavior, and physiology of the original tissue (Sato et al., 2009). Like traditional cell lines, organoids are amenable to genomic, transcriptomic, and proteomic analyses, as well as high-throughput drug screening. Tumor organoids have emerged as powerful tools in cancer research, offering a platform for personalized medicine by accurately predicting patient-specific drug responses (Yang et al., 2023; Roerink SF, et al., 2018; Vlachogiannis et al., 2018; Ooft et al., 2019; Yao et al., 2020).

In this study, we used an *in vitro* colon organoid model to investigate the molecular mechanisms underlying regorafenib resistance in the epithelium. Organoids were derived from mouse colon tissue expressing or lacking the tumor suppressor gene *Apc*, which is mutated in most sporadic CRC (Fodde, 2002). Through transcriptomic, proteomic and cellular analyses, we found that regorafenib resistance involves an EMT-associated mechanism. Importantly, co-treatment with an autophagy inhibitor enhanced sensitivity to regorafenib. These findings, which were also validated in human CRC cell lines, suggest that combination therapy may offer a promising strategy to overcome chemoresistance in mCRC patients.

## 2 Materials and methods

### 2.1 Mice

All animal procedures complied with European Union guidelines and were approved by the local ethics committee (CEBEA from the faculty of Medicine, ULB) under the accepted protocol 631N. To generate tumor-derived organoids, *Tg(Vil1-cre/ERT2)23Syr/J* (El Marjou et al., 2004) and *Apc* ^*tm1Tyj/J*^ (referred to as Apc^flox^) (Robanus-Maandag et al., 2010) mice were bred and maintained under pathogen-free conditions. Adult *Vil-cre/ERT2/Apc* ^*wt/wt*^ and *Vil-cre/ERT2/Apc* ^*flox/flox*^ mice were intraperitoneally injected with tamoxifen (2 mg per 30 g of body weight) for three consecutive days to induce recombination at the *Apc* locus. Colon tissues were harvested 2–3 days after the final injection. Tamoxifen was dissolved in a sunflower oil/ethanol mixture (9:1) at 10 mg/mL (Sigma-Aldrich and VWR, respectively). Colon fibroblast cells were obtained from C57BL/6 mice (Janvier-Labs).

### 2.2 Cell culture procedures

#### 2.2.1 Mouse colon organoid cultures

To generate colon organoids, adult mouse colon was cut into 3–5 mm pieces and incubated in 10 mM EDTA (Invitrogen) in DPBS (Gibco) for 30 min on ice with shaking at 80 rpm. Mechanical dissociation was performed by ups-and-downs in a fetal bovine serum (FBS, Thermofisher) pre-coated 10 mL pipette. The suspension was filtered through a 70 µm filter (Corning) and centrifuged at 300 × g for 5 min. Pelleted crypts were embedded in LDEV-free Matrigel (Corning) and incubated for 20 min at 37°C before adding culture medium, as described (Sato et al., 2011). The culture medium consisted of Advanced-DMEM/F12 medium (Gibco) supplemented with 20 mM GlutaMAX (Gibco), 1X N2 (Gibco), 1X B27 w/o vit. A (Gibco), penicillin-streptomycin, gentamycin and amphotericin cocktail, 10 mM HEPES (all from Thermofisher Scientific), 1 mM N-acetyl cysteine and 10 mM nicotinamide (both from Sigma-Aldrich), 50 ng/mL EGF, 100 ng/mL Rspondin 1 (both from R&D systems), 100 ng/mL Noggin (Peprotech), and 50% Wnt3a conditioned medium produced with L Wnt-3A cells (ATCC CRL-2647) following manufacturer’s instructions. Culture medium was changed every other day and after 8–9 days in culture, organoids were harvested and digested with TripLE Express (Thermo Fisher Scientific) for 5 min at 37°C. Cells were centrifuged at 1,300 rpm for 5 min and (re)plated in Matrigel as described above. Culture media were supplemented with 10-µM Y-27632 (Sigma-Aldrich) during initial seeding and replating for the first 48 h. See Supplementary Table S1 for organoid lines characteristics. Quantitative image analysis of vesicles in organoids was performed using QuPath (Figure 2E). For each image, a region corresponding to an on-focus area within the organoids was delineated. The area of each region was measured in pixels, and the number of visible vesicles within each was manually quantified.

#### 2.2.2 Mouse colon fibroblast cultures

Mouse fibroblasts isolated from C57B/6 mice were isolated from residual colon tissue following crypt enrichment (see section 2.2.1). Tissue fragments were washed in DMEM/F-12 supplemented with 10% FBS and 2% penicillin/streptomycin by manual shaking and centrifugation (1,300 rpm, 4 min). This step was repeated three times to remove debris and loosely attached cells. Tissue fragments were then digested in DMEM/F-12 containing 10% FBS, 1% penicillin/streptomycin, 1 mg/mL Collagenase D (Sigma-Aldrich) and 1 mg/mL Dispase (Gibco) at 37°C for 45 min with agitation and manual pipetting every 15 min to facilitate tissue dissociation. After digestion, samples were centrifuged at 1,300 rpm for 4 min, and the cell pellet was resuspended in DMEM with 10% FBS, 1% penicillin/streptomycin, and 1% amphotericin. The suspension was filtered through a 100 μm strainer and seeded into 6-well plates. See Supplementary Table S1 for fibroblast cell lines characteristics.

#### 2.2.3 CRC cell cultures

The commercially available human CRC cell lines were cultured in a 5% CO_2_ incubator at 37°C with the following media: HT-29 (ATCC, #HTB-38™) and HCT 116 (ATCC, #CCL-247™) in Mc Coy medium (VWR), DLD-1 (ATCC, #CCL-221™) and LoVo (ATCC, #CCL-229™) in RPMI (Gibco), and SW480 (ATCC, #CCL-228™) in EMEM (ATCC). All media were supplemented with 10% FBS and 1% penicillin-streptomycin. See also Supplementary Table S1.

#### 2.2.4 Inhibitors

Regorafenib (Selleckchem.com) and autogramin-2 (MedChemExpress) were prepared as 10 mM stock solutions in DMSO. They were used on organoids, CRC and fibroblast cells at concentrations and for duration times that are indicated in Figures and Figures legends.

### 2.3 Cell viability assay

CRC cell lines and fibroblasts were seeded at densities of 10,000 and 30,000 cells per well, respectively. CRC lines were seeded in poly-L-lysine (Sigma-Aldrich)-coated 96-well plates. After 24 h, cells were incubated with compounds at the indicated concentrations. Following 72 h of treatment, the medium was removed, and cells were rinsed with phosphate-buffered saline (PBS), fixed with 10% neutral buffered formalin (NBF) solution (Avantor) for 15 min and stained with 0.1% crystal violet (Sigma-Aldrich) for 30 min. Wells were washed with tap water and lysed with 0.2% Triton X-100 in DPBS (Sigma-Aldrich) for 90 min. Absorbance was measured at 570 nm using a BIO RAD iMark Microplate Reader.

### 2.4 Tissue processing, immunohistochemistry and immunofluorescence

Samples were fixed with 10% NBF solution for 20 min at room temperature, followed by sequential sedimentation through 20% and 30% sucrose solutions before embedding in Tissue freezing medium (Leica). Section (6 µm) were used for immuno-fluorescence, histochemistry and *in situ* hybridization. For immunofluorescence/histochemistry, antigen retrieval was performed using 10 mM sodium citrate. Organoid slides were incubated with primary antibodies overnight at 4°C. Detection was performed using HRP-conjugated secondary antibodies (Jackson Laboratories), ABC kits and DAB substrate (Vector Labs), followed by hematoxylin counterstaining (Millipore). For immunofluorescence, fluorochrome-conjugated secondary antibodies and DAPI were used. Slides were mounted with Coverquick 4000 (VWR Chemicals) for histochemistry or Fluorsave for fluorescence (Millipore). *In situ* hybridization was performed using the RNAscope kit (ACD-Biotechne) according to manufacturer’s instructions. Imaging was performed using a Nanozoomer digital scanner S360 for brightfield or a Zeiss Axio Observer inverted microscope with Zen Pro software for fluorescence. Antibodies, RNAscope probes and assay kits are listed in Supplementary Table S2.

### 2.5 RNA extraction, RNA sequencing and gene set enrichment analysis (GSEA)

**FIGURE 1:**
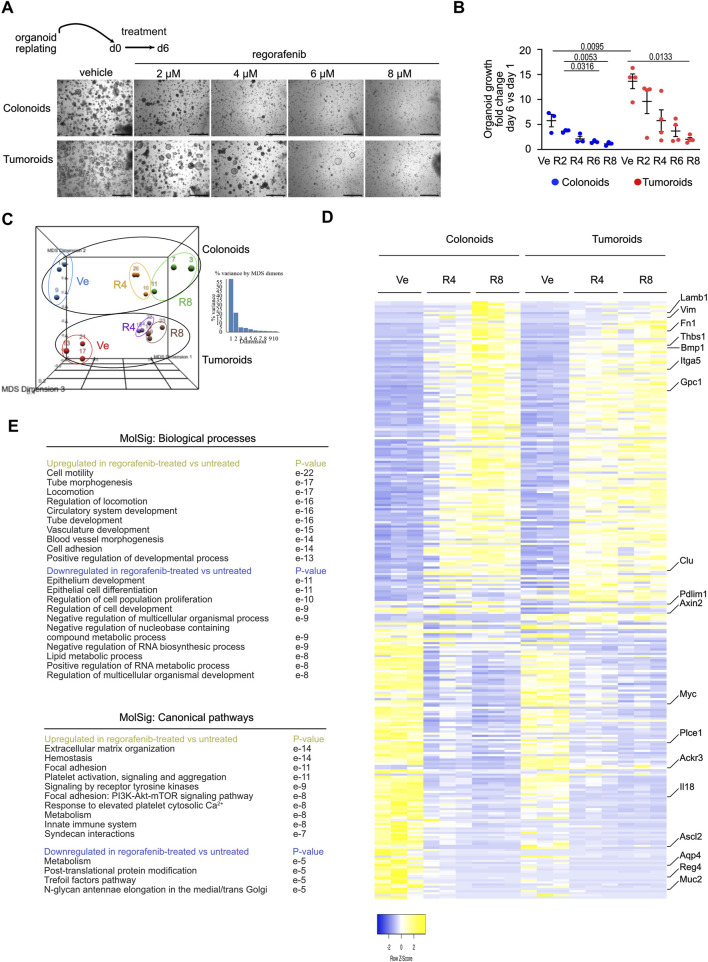
Regorafenib activates an Epithelial Mesenchymal Transition process in mouse organoids **(A)**. Representative pictures showing the effect of regorafenib concentration on mouse colonoid or tumoroid cultures at day 6 (d6), endpoint of the treatment. Scale bars: 500 µm. **(B)** Quantification of organoid growth estimated as the mean area fold change of day 6 versus (vs.) day 1. Ve: Vehicle, 2 µM (R2), 4 µM (R4) or 8 µM (R8) regorafenib. Each symbol refers to an individual organoid line generated from an individual mouse (n = 3 colonoids, n = 4 tumoroids). One hundred elements were analyzed per organoid line. Data are represented as means ± sem. Two-way ANOVA tests with Tukey’s multiple comparisons. **(C)** Principal component analysis (PCA) plot of vehicle (Ve), 4 µM (R4) or 8 µM (R8) regorafenib-derived organoid transcriptomes. Each dot refers to an individual sample (n = 3 colonoid and 3 tumoroid lines). Fold change relative to colonoids in Ve conditions with false discovery rate of 0.001 and absolute fold change of 0.585 (11,308 expressed genes). **(D)** Heatmap of the 277 differentially expressed genes in vehicle (Ve), 4 µM (R4) or 8 µM (R8) regorafenib-derived organoids (log 2-fold change). **(E)** GSEA-Biological processes and Canonical pathways for upregulated and downregulated gene lists in regorafenib vs. vehicle-treated organoids. p value is indicated.

Total RNA from organoids and fibroblasts was extracted using Rneasy mini kit (Qiagen) for crypts and cell lines following manufacturer’s instructions. RNA quality was assessed using a Fragment analyzer 5200 (Agilent technologies). RNA samples from organoids were used to generate indexed cDNA libraries using the NEB Next Ultra II directional RNA Library Prep Kit for Illumina® E7760L (NEW ENGLAND BioLabs Inc) according to the manufacturer’s protocol. Multiplexed libraries were sequenced on a NovaSeq 6000 (Illumina) using an S2 flow cell and sequences were produced using a 200 Cycles Kit. Sequencing reads were trimmed for adaptor sequence (Trimmomatic-0.36). After transcripts assembling, gene-level counts were obtained using HTSeq-0.9.1. Paired-end reads were aligned to the mouse reference genome GRCm38 using STAR, and annotation was based on the Mus_musculus.GRCm38.90.gtf file obtained from ftp.Ensembl.org. Differential gene expression was conducted using the Degust tool from Monash University (Powel, 2019). CSV files containing gene expression data (based on 20 million mapped reads) were uploaded, and differentially expressed genes were identified using the EdgeR quasi-likelihood method with the following criteria: a minimum of 10 CPM (count per million) in at least two samples. Biological and canonical processes enriched in the output lists of upregulated and downregulated genes were further investigated using the molecular signatures database GSEA MolSig (Broad Institute) with the FDR q value set to less than 0.05 (Subramanian et al., 2005). Heatmaps in Figures 1D, 6D were generated by uploading the list of differentially expressed genes obtained from Degust onto the Heatmapper web server (Babicki et al., 2016).

### 2.6 Gene expression analysis by qPCR

qRT-PCR experiments were performed on total RNA extracted from tumoroids using the RNeasy Mini Kit (Qiagen). A DNAse I treatment (Invitrogen) was used to remove potential DNA contaminants. cDNA was prepared using RnaseOUT and Superscript II according to the manufacturer’s protocol (Invitrogen). qPCRs were performed on the qTower 3 from Analytik Jena. Gene expression levels were normalized to *Ywhaz* used as a reference gene and quantified using the qBase Software (Biogazelle). Primer sequences are listed in Supplementary Table S2.

### 2.7 Proteomic profiling

For Western blot analysis, colon organoids were treated with compounds for 6 days. Organoids were collected using Cell Recovery Solution (Corning), incubated for 1 h to dissolve Matrigel, and washed twice with PBS. Organoids were lyzed in 100 μL of buffer containing a protease inhibitor cocktail (Roche). The lysis buffer consisted of HEPES (50 mM), NaCl (150 mM), EDTA (10 mM), sodium pyrophosphate (10 mM), sodium fluoride (100 mM), and sodium orthovanadate (Na_3_VO_4_, 2 mM) in Milli-Q water, with the pH adjusted to 7.5. Ten and thirthy micrograms of proteins per organoid and CRC line sample, respectively, were loaded onto 15% polyacrylamide SDS-PAGE gels and transferred to nitrocellulose membrane. Membranes were blocked in 5% milk at room temperature for 1 h, and incubated overnight at 4°C with phospho-p44/42 MAPK primary antibody. After three washes in PBS 0,01% Tween (BioRad), membranes were incubated at room temperature for 1 h with IgG HRP-conjugated goat anti-rabbit secondary antibody diluted in 5% milk. Chemiluminescent detection was performed using a 1:1 mixture of substrate (ThermoFisher). Membranes were stripped using 0.1 M glycine (pH 2.8) on a shaking platform for 30 min, repeated four times. To neutralize the pH, membranes were washed twice in PBS for 5 min each. Subsequently, membranes were re-blocked at room temperature for 1 h in 5% milk, followed by overnight incubation at 4°C with p44/42 MAPK primary antibody. The rest of the procedure was as described above. Antibodies are listed in Supplementary Table S2. For secretome analysis, culture supernatants from organoids treated for 6 days were collected, centrifuged at 300 *g* for 15 min and stored at −20°C until use. For each sample, 200 μg of proteins were loaded onto Mouse XL Cytokine Array membranes (R&D Systems) according to the manufacturer’s instructions. For proteome profiler phospho-kinase assays (R&D Systems), organoids were harvested using the Cell recovery solution and 280 μg of proteins were loaded onto membranes. Protein concentration was determined using the Pierce BCA Protein Assay kit (ThermoFisher). Images were captured using the Solo S imaging system (Vilber Lourmat) at various exposure times.

### 2.8 Statistical analysis

Statistical analyses were performed with Graph Pad Prism version 10. All experimental data are expressed as mean ± sem unless otherwhile stated in Figure legends. The number of biological replicates used for each experiment is reported in Figure legends. The significance of differences between groups was determined by appropriate parametric or non-parametric tests as described in Figure legends. In all cases P < 0.05 was considered statistically significant. Exact p-values are reported in the Figures.

### 2.9 Data availability

The data sets generated and analyzed during the current study are available in the GEODATASET repository [GSE297312.]. The pwad038 Supplementary Table S3 from Mao et al (2024) was used to compare the regorafenib-induced gene expression signature (Pattern G5) in human CRC tumor-derived organoids treated with the data obtained in this study.

## 3 Results

### 3.1 Regorafenib activates an Epithelial Mesenchymal Transition program in mouse organoids

To better understand the molecular mechanisms underlying tumor resistance to regorafenib, a multi-kinase inhibitor currently used in CRC patients, mouse colon organoid lines were established from tamoxifen-treated VilCreERT2/Apc^+/+^ and VilCreERT2/Apc^fx/fx^ mice, generating colonoids and tumoroids, respectively. After replating and expansion, organoid lines were seeded in Matrigel and treated for 6 days with different concentrations of regorafenib or vehicle control (0.5% DMSO, corresponding to the vehicle concentration at the highest regorafenib dose). At this concentration, DMSO did not significantly alter gene expression compared to standard Sato culture medium (Supplementary Figure S1A). Notably, 8 µM regorafenib corresponds to the average plasma concentration observed in treated patients (EMA, 2025). After 6 days of treatment, regorafenib induced a dose-dependent reduction in growth in both colonoids and tumoroids, despite the observed growth advantage conferred by Wnt pathway activation in tumoroids under control conditions (Figures 1A,B). To investigate the underlying mechanisms, organoids treated with regorafenib or vehicle for 6 days were harvested for bulk RNA sequencing. As shown in Figure 1C, the transcriptomes of colonoids and tumoroids were clearly distinguishable. Under control conditions, 70 genes were differentially expressed between tumoroids and colonoids using a false discovery rate (FDR) of 0.01 and an absolute log2-fold change ≥1 (Figures 1C,D). Tumoroids demonstrated upregulation of oncogene-associated genes (*Ackr3*, *Adcy3*, *Fam222*) and downregulation of tumor suppressor genes (*Casz1*, *Frk*, *Homer2*, *Pdlim1*) (Supplementary Figure S1B). As expected, tumoroids also showed elevated expression of intestinal stem cell signature genes (*Axin2*, *Ascl2*, *Myc*, *Tiam1*, *Cdc7c)* from Muñoz et al. (2012) due to Apc loss-of-function (Supplementary Figure S1C). Regorafenib treatment led to 277 differentially expressed genes in organoids (i.e., both colonoids and tumoroids) using a FDR 0.001 and log2-fold change ≥0.585 (Figures 1C,D). Treatment upregulated genes involved in cell motility, tube morphogenesis, locomotion, circulatory system development (e.g., *Thbs1*, *Bmp1, Gpc1*, *Fn1*), while downregulating genes associated with epithelial development, epithelial cell differentiation, regulation of cell population proliferation and negative regulation of nucleobase containing compound metabolic process (e.g., *Chga*, *Muc2*, *Il18*) (Figures 1D,E; Supplementary Figure S1C). Markers of the intestinal stem cell signature (*Ascl2*, *Myc*, *Tiam1*, *Cdc7*, *Aqp4*, *Plce1*) were also downregulated in both organoid types (Supplementary Figure S1C). Collectively, these data indicate that regorafenib treatment activates an EMT process and suggest overall reduced stem cell activity and epithelial proliferation.

### 3.2 Phospho-ERK signaling activated by regorafenib treatment is attenuated by autophagy inhibition in mouse organoids

**FIGURE 2:**
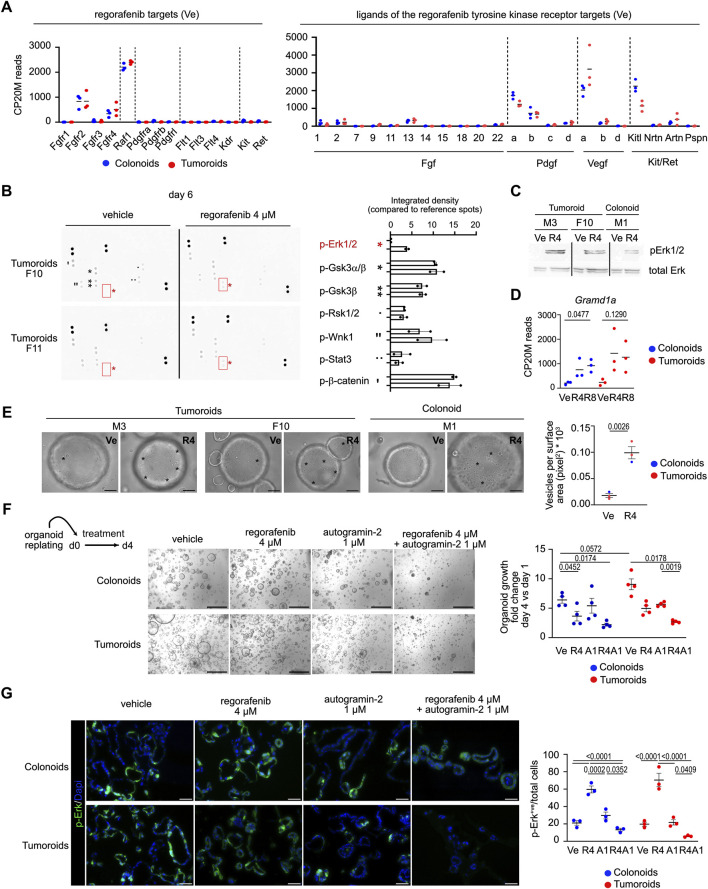
Phospho-ERK signaling activated by regorafenib treatment is suppressed by autophagy inhibition in mouse organoids **(A)**. Expression levels of genes coding for regorafenib targets and for ligands of the tyrosine kinase receptors in Vehicle (Ve) culture conditions. CP20M: counts per kilobase of transcript per 20 million mapped reads. Each symbol corresponds to the value of an organoid line generated from an individual mouse. **(B)** Membranes showing protein kinase phosphorylation in vehicle or regorafenib-treated tumoroids from two different lines. Right panel: integrated density for the detected signals. **(C)** Western blot showing phospho-Erk (p-Erk1/2) induction in regorafenib 4 µM (R4) vs. vehicle (Ve)-treated tumoroid and colonoids. **(D)** Expression levels of the *Gramd1a* gene. CP20M: counts per kilobase of transcript per 20 million mapped reads. Vehicle (Ve), 4 µM regorafenib (R4) or 8 µM regorafenib (R8). Each symbol corresponds to the value of an organoid line generated from an individual mouse. One way ANOVA test with Tukey’s multiple comparisons. **(E)** Representative brightfield pictures showing the presence of vesicles (evidenced by asterisks) in 4 µM regorafenib (R4)-treated organoids compared to vehicle (Ve) conditions at day 4. Right panel: quantification of the vesicular density as the number of vesicles per surface area. Ten elements were analyzed per organoid line per culture condition. Unpaired t-test. Scale bar: 50 µm. **(F)** Representative pictures showing the effect of regorafenib, autogramin-2 or combined treatment on mouse colonoid or tumoroid cultures at day 6 (d6), endpoint of the treatment. Scale bars: 500 µm. Right panel: quantification of organoid growth estimated as the mean area fold change of day 4 versus (vs.) day 1. Vehicle (Ve), 4 µM regorafenib (R4), autogramin-2 1 µM (A1) and combined treatment (R4A1). Each symbol refers to an individual organoid line generated from an individual mouse (n = 4 colonoids, n = 4 tumoroids). One hundred elements were analyzed per organoid line. Data are represented as means ± sem. Two-way ANOVA tests with Tukey’s multiple comparisons. **(G)** Representative pictures of immunofluorescence showing phospho-Erk levels in vehicle or treated colonoids and tumoroids. Nuclei counterstained with Dapi. Scale bars: 50 µm. Right panel: quantification of p-Erk positive (+ve) cells relative to the total number of cells. Vehicle (Ve), 4 µM regorafenib (R4), autogramin-2 1 µM (A1) and combined treatment (R4A1). Each symbol refers to an individual organoid line (n = 3 colonoids, n = 3 tumoroids). An average of 850 cells were analyzed per organoid line. Two-way ANOVA tests with Tukey’s multiple comparisons.

To investigate the pathway(s) involved in the organoid response to regorafenib, expression of known drug targets was first assessed under control conditions (Figure 2A, left panel). Among the tyrosine kinase receptors targeted by regorafenib, *Fgfr2*, and to a lesser extent *Fgfr4*, were expressed, along with the intracellular kinase *Raf1*. The only ligands detected for the tyrosine kinase receptors were *Fgf2/13*, *Pdgfa/b*, *Vegfa/b* and *Kitl*; suggesting that regorafenib primarily inhibits intracellular Raf1 activity in cultured organoids (Figure 2A, right panel). Then, signaling cascades modulated by regorafenib were examined in two tumoroid lines using proteome profiler phospho-arrays (Figure 2B). While phosphorylation levels of β-catenin, GSK3α/β, and Wnk1 (detected at baseline) were not affected by drug treatment, regorafenib induced phosphorylation of Erk1/2 (Figures 2B,C). Although Erk1/2 activation via phosphorylation typically occurs in response to mitogenic signals via receptor tyrosine kinases, it can also been triggered by environmental stressors such as chemotherapy, through a process involving autophagy (Bishnu et al., 2021). Interestingly, *Gramd1a,* which encodes a cholesterol transfer protein required for autophagosome biogenesis (Laraia et al., 2019), was upregulated following regorafenib challenge in both colonoids and tumoroids (Figure 2D). Brightfield imaging revealed a marked increase in vesicle formation in regorafenib-treated organoids compared to controls, consistent with active autophagy (Figure 1E). To assess the role of autophagy in the regorafenib response, organoids were treated with autogramin-2, an autophagy inhibitor targeting GramD1A, at 1 μM, either alone or in combination with 4 µM regorafenib for 4 days (Laraia et al., 2019). The combination significantly reduced organoid growth compared to regorafenib alone (Figure 2F) and was associated with a marked decrease in the proportion of phospho-Erk1/2 positive (+ve) cells in both kind of organoids (Figure 2G). Moreover, cotreatment reduced the density of LC3B^+ve^ labeled autophagosomes compared to regorafenib or autogramin-2 treatments alone (Supplementary Figure S2). Of note, consistent with high basal autophagy levels reported in intestinal and colon stem/progenitor cells (Groulx et al., 2012), organoids exhibited high density of LC3B^+ve^ punctuate staining in vehicle conditions (Supplementary Figure S2).

### 3.3 Autophagy inhibition improves regorafenib-mediated effect on mouse organoids

**FIGURE 3:**
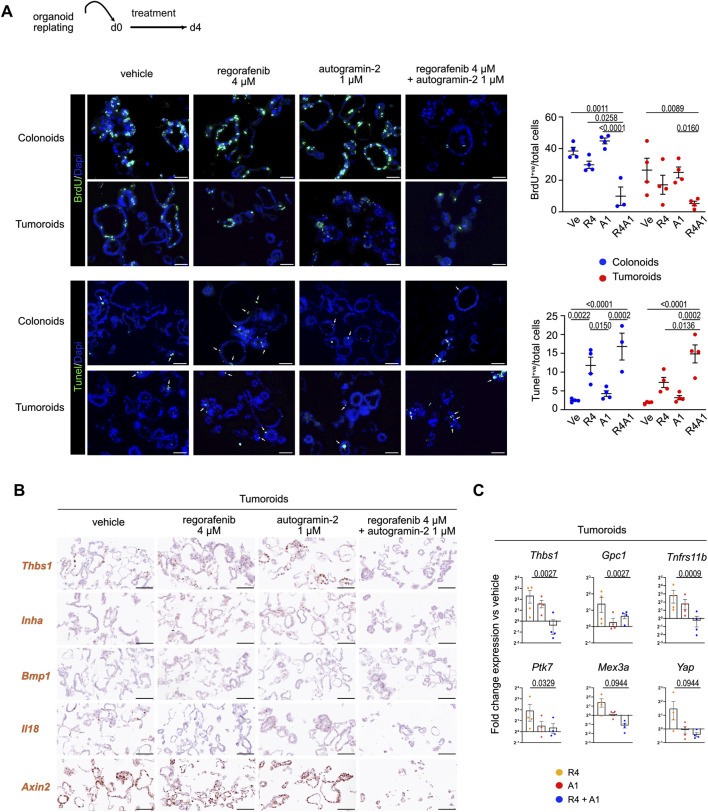
Combined regorafenib treatment and autophagy inhibition reduces the EMT-related chemoresistance process in mouse organoids **(A)**. Representative pictures of immunofluorescence showing cell proliferation (BrdU incorporation) and cell apoptosis (Tunel assay) in vehicle or treated colonoids and tumoroids. Nuclei counterstained with Dapi. Arrows indicate apoptotic cells. Scale bars: 50 µm. Right panels: quantification of BrdU^+ve^ cells or Tunel^+ve^ cells relative to the total number of cells. Each symbol refers to an individual organoid line (n = 4 colonoids, n = 4 tumoroids). Vehicle (Ve), 4 µM regorafenib (R4), autogramin-2 1 µM (A1) and combined treatment (R4A1). An average of 750 cells were analyzed per organoid line. Two-way ANOVA tests with Tukey’s multiple comparisons. **(B)** Expression of EMT-related transcripts detected in tumoroids by RNAscope. Scale bars: 100 µm. **(C)** Gene expression analysis by qRT-PCR of the indicated genes modulated by single or combined drug challenge. 4 μM regorafenib (R4), autogramin-2 1 µM (A1) and combined treatment (R4A1). Each symbol corresponds to a given tumoroid line. Expression levels are relative to vehicle-treated sample set at 1 per tumoroid line. One-way ANOVA tests with Dunn’s multiple comparisons. *Thbs1*: R4 vs. R4A1: p = 0.0370; *Gpc1*: R4 vs. R4A1: p = 0.0370; *Tnfrs11b*: R4 vs. R4A1: p = 0.0155; *Ptk7*: Ve vs. R4 and R4 vs. R4A1: p = 0.0823; *Mex3a*: R4 vs. R4A1: p = 0.0823; *Yap*: R4 vs. R4A1: p = 0.0823.

We next investigated the impact of single or combined treatments on cell proliferation and programmed cell death in organoids. While treatment with autogramin-2 alone barely affected either cell proliferation or apoptosis compared to vehicle controls, co-treatment with regorafenib and the autophagy inhibitor enhanced the anti-proliferative effect of regorafenib and increased apoptosis (Figure 3A). Moreover, the impact of cotreatment on the expression of various genes differentially modulated upon regorafenib treatment was studied by *in situ* hybridization and qRT-PCR experiments (Figures 3B,C). Among the most upregulated genes in regorafenib-treated samples was *Thbs1,* which encodes the matricellular protein Thrombospondin-1. This protein is reported to mediate non-cell-autonomous morphological and transcriptional responses in Apc-deficient intestinal organoids, promoting activation of the surrounding normal epithelium (Jacquemin et al., 2022). *Thbs1*, which is also overexpressed in the stromal compartment of colon tumors, is thought to contribute to immunosuppression in CRC (Omatsu et al., 2023). Co-treatment with regorafenib and autogramin-2 significantly reduced *Thbs1* expression compared to vehicle-treated conditions (Figures 3B,C). A similar expression pattern was detected for *Inha*, which encodes Inhibin A, a protein implicated in 5-FU resistance in colon cancer cells (Zhang et al., 2024), and, to a lesser extent, the *Gpc1*, a gene associated with EMT activation, increased invasion and migration in CRC cells, and proposed as a biomarker for stage III CRC relapse (Li et al., 2017). Furthermore, genes such as *Ptk7* (overexpressed in colon carcinoma cells), *Mex3a* (a marker of drug-tolerant persister CRC cells) and *Yap* (involved in 5-FU resistance in CRC cell lines), were upregulated following regorafenib treatment alone but returned to baseline levels whith combination treatment (Figure 3C) (Jin et al., 2024; Álvarez-Varela et al., 2022; Xu et al., 2024). However, *Bmp1*, which was upregulated by regorafenib, was not significantly affected by the addition of autogramin-2 (Figure 3B). Moreover, *Il18*, which encodes a proinflammatory cytokine that may enhance anti-tumor ability of natural killer cells against CRC, was among the most downregulated genes following regorafenib treatment, alone or in combination with autogramin-2 (Figure 3B) (Li et al., 2021).

**FIGURE 4:**
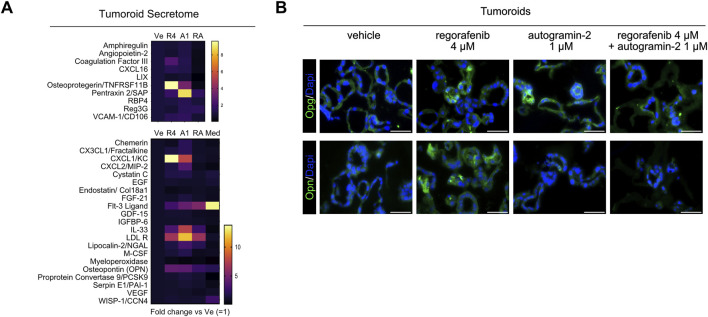
Secretome analysis of tumoroids **(A)**. Heatmap showing tumoroid secreted molecules upon 4 µM regorafenib (R4), autogramin-2 1 µM (A1), combined drug treatment (RA) vs. vehicle (Ve) or culture medium alone (Med). Mean of two independent (Ve, R4 and RA) or a single experiment (A, Med). **(B)** Representative pictures of immunofluorescence showing expression of Osteoprotegerin/Tnfrsf11b (OPG) and Osteopontin/Spp1 (OPN) in vehicle or drug-treated tumoroids. Nuclei counterstained with Dapi. Scale bars: 50 µm.

Next, to investigate potential changes in the secretome profile of tumoroids following drug treatment, we performed cytokine assays to screen for 70 secreted molecules (Figure 4A). Thirty-one proteins were detected at significant levels in culture supernatants across two independent experiments. Of these, 10 proteins were only detected in tumoroid cultures, while 21 were present in the culture medium regardless of the presence of cells (Figure 4A). As shown in the heatmaps representing linear fold changes relative to vehicle-treated conditions, Osteoprotegerin (*Tnfrs11b/Opg*) was the most differentially secreted protein following regorafenib treatment. Notably, co-treatment with autogramin-2 abolished its secretion. These data are consistent with qRT-PCR and immunofluorescence data, which also showed downregulation of *Tnfrs11b* mRNA levels and loss of protein expression in tumoroids upon cotreatment (Figures 3C, 4B). Interestingly, Osteoprotegerin has been proposed as a prognostic marker in CRC and has been shown to suppress memory CD4^+ve^ T cell infiltration (Zhang et al., 2021). Moreover, Cxcl1(KC) and Osteopontin (Opn/Spp1) were secreted in response to regorafenib treatment, whereas cotreatment reduced their levels (Figure 4A). Expression of Opn was confirmed by immunofluorescence staining of tumoroid sections using anti-Opn antibodies (Figure 4B). Altogether, these experiments with mouse organoids revealed that regorafenib induces transcriptomic and proteomic changes associated with EMT and chemoresistance, which can be effectively mitigated by cotreatment with the autophagy inhibitor autogramin-2.

### 3.4 Autophagy inhibition can improve sensitivity of CRC lines to regorafenib

**FIGURE 5:**
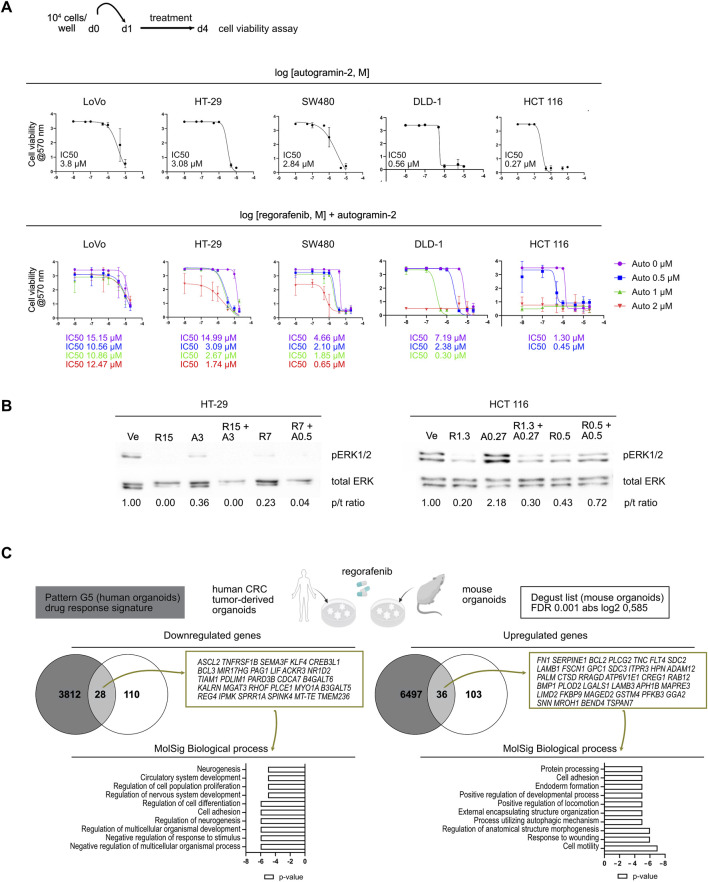
Autophagy inhibition improves sensitivity to regorafenib in colorectal cancer cell lines **(A)**. Dose-response curves of colorectal cancer cell lines treated with autogramin-2 (upper panels) or regorafenib combined with various concentrations of autogramin-2 (lower panels). The schematic representation of the cell viability assays performed on cell lines is shown. The IC50 for autogramin-2 is indicated in the graph for each cell line. Dose response curves for autogramin-2 were generated from the mean of 4 (LoVo) or 3 (HT-29, SW480, HCT 116, DLD-1) independent experiments, each performed in triplicate. Dose response curves for combined treatment were generated from the mean of 2 (LoVo, DLD-1) or 3-5 (HT-29, SW480, HCT 116) independent experiments, performed in triplicate. IC50 curves are represented as the mean ± sd of independent experiments. **(B)** Western blots of HT-29 and HCT 116 cells treated with vehicle (Ve), regorafenib (R) and autogramin-2 (A) at the indicated concentrations (µM). p/t ratio refers to the ratio of p-ERK 1/2/total ERK signals. Pictures represent a single experiment. **(C)** Compared gene expression profiles of mouse colon organoids (the 277 gene list) and human CRC tumor-derived organoids [drug response signature pattern (G5) reported by Mao et al (2024)] induced by regorafenib treatment. The genes commonly upregulated and downregulated in both types of samples were identified using the Venny 2.0.2 tool and the associated biological processes were further analyzed by GSEA MolSig. A list of common genes is provided.

We extended our analysis to several high-grade human CRC cell lines representing CMS1 (LoVo, DLD-1), CMS3 (HT-29) and CMS4 (SW480, HCT 116) subtypes, following the protocol outlined in Figure 5A (Berg et al., 2017; Luk et al., 2025). Dose-response curves for autogramin-2 revealed different sensitivities, with IC50 values ranging from 3.8 µM in LoVo cells to 0.27 µM for HCT 116 cells (Figure 5A, upper panels). Then, dose-response assays were performed using regorafenib alone or in combination with 3 concentrations of autogramin-2 (Figure 5A, lower panels). At the lowest tested concentration (0.5 µM), autogramin-2 significantly reduced the IC50 of regorafenib in 4 out of 5 cell lines. LoVo cells, however, appeared relatively resistant to both single and combination treatments (Figure 5A). These data suggested that autophagy inhibition can enhance regorafenib sensitivity in human CRC cells. To further explore the underlying mechanisms, we analyzed pERK 1/2 signaling by Western blot. As expected, based on their mutational profiles, HT-29 (BRAF V600E) and HCT116 (KRAS G13D) cells, exhibited constitutive ERK activation under vehicle conditions (Figure 5B). Regorafenib treatment reduced pERK 1/2 signals in both lines. In HT-29 cells, autogramin-2 alone downregulated ERK signaling and acted synergistically with regorafenib (Figure 5B, left panels). In contrast, HCT 116 cells, highly sensitive to autogramin-2, showed an unexpected increase in pERK 1/2 levels upon treatment with the autophagy inhibitor, either alone or in combination with regorafenib (Figure 5B, right panels). These differences may reflect distinct mutational landscapes, and further experiments are needed to fully elucidate the signaling dynamics involved. Finally, to assess the translational relevance of our findings, we compared the regorafenib response-associated gene signature (referred to as pattern G5) reported by Mao et al. (2024) with the list of genes modulated in mouse colon organoids following regorafenib treatment (Figure 5C). Twenty-five percent of the mouse genes overlapped with the human G5 signature. Interestingly, regorafenib commonly induced genes involved in “cell motility”, “response to wounding”, and “positive regulation of locomotion”, consistent with EMT activation. Genes associated with “process utilizing autophagic mechanism” were also upregulated, reinforcing the role of autophagy in drug resistance (Figure 5C). Conversely, downregulated genes were enriched in processes such as “negative regulation of multicellular organismal process, regulation of cell differentiation, regulation of cell population proliferation” (Figure 5C). Together, these analyses confirmed that regorafenib treatment downregulates stem cell and differentiation markers and implicates EMT and autophagy as key components of the resistance mechanism.

### 3.5 Poor sensitivity of mouse primary fibroblasts to combined regorafenib and autogramin-2 treatment

**FIGURE 6:**
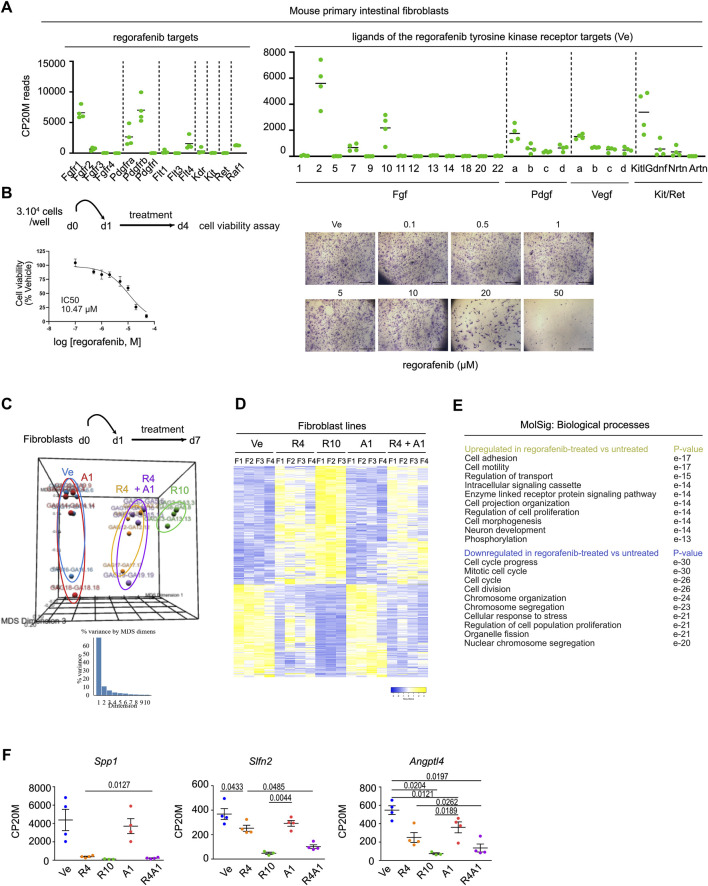
Mouse colon fibroblasts exhibit higher resistance to regorafenib treatment and autophagy inhibition **(A)**. Expression levels of genes coding for regorafenib targets and ligands of the tyrosine kinase receptors. CP20M: counts per kilobase of transcript per 20 million mapped reads. Each symbol corresponds to the value of a fibroblast cell line generated from an individual mouse. **(B)**Scheme of cell viability assays performed on mouse colon fibroblast cell lines. Left panel: dose-response curve of fibroblast cell lines treated with regorafenib (mean ± sd). The IC50 is indicated in the graph. Dose response curve was generated from mean values of 7 independent lines tested in triplicates, each line being generated from an individual mouse. Right panels: cell viability assay in one representative fibroblast cell line. Scale bars: 600 µm. **(C)**Principal component analysis (PCA) plot of vehicle (Ve), 1 µM autogramin-2 (A1), 4 µM (R4), 10 µM (R10) regorafenib or combined (R4A1)-treated fibroblast transcriptomes. Each dot refers to an individual sample (n = 4 fibroblast cell lines). Fold change relative to fibroblasts in Ve conditions with false discovery rate of 0.05 and absolute fold change of 0.585 (11,025 expressed genes). **(D)**Heatmap of the 630 differentially expressed genes in vehicle (Ve), 1 µM autogramin-2 (A1), 4 µM (R4), 10 µM (R10) regorafenib or combined (R4A1)-treated fibroblast transcriptomes (log 2-fold change). **(E)**GSEA- Biological processes for upregulated and downregulated gene lists in drug-treated vs. vehicle-treated organoids. p value is indicated. **(F)**Expression levels of several genes downregulated by regorafenib treatment. CP20M: counts per kilobase of transcript per 20 million mapped reads. vehicle (Ve), 1 µM autogramin-2 (A1), 4 µM (R4), 10 µM (R10) regorafenib or combined (R4A1). Each symbol corresponds to the value of an organoid line generated from an individual mouse. One-way ANOVA tests with Tukey’s multiple comparisons.

To explore the effect of regorafenib on the stromal compartment, we established primary fibroblast cell lines from the colons of C57B/6 mice. First, bulk RNA sequencing revealed the presence of all fibroblast subtypes under standard culture conditions, with *Pdgfra* serving as a general marker (Brügger and Basler, 2023). Colonic “crypt fibroblast” subtypes 1 and 2 (*Cd34*/*Cd90*-positive) as well as colonic “top fibroblasts” were detected with expression of the specific *Col15a1*/*Sfrp1* (colonic crypt subtype 1), *Cd81*/*Grem1*/*Rspo3* (colonic crypt subtype 2) and *Tnc*/*Procr*/*Wnt5a* (colonic top) markers (Supplementary Figure S3). Cultured fibroblasts expressed several known targets of regorafenib, including *Fgfr1*, *Pdgfra, Pdgfrb*, *Flt4* and *Raf1*, along with ligands from the FGF, PDGF and VEGF pathways, suggesting potential effect of regorafenib treatment on these cells through multiple signaling routes (Figure 6A). However, cell viability assays performed on fibroblasts revealed an IC50 of 10.47 µM, indicating that fibroblasts are relatively resistant to regorafenib at clinically relevant concentrations (Figure 6B). We next compared the transcriptomes of fibroblasts treated with vehicle, autogramin-2 (1 µM), regorafenib (4 or 10 µM) or a combination of autogramin-2 (1 µM) and regorafenib (4 µM). Using an FDR of 0.05 and an absolute log2-fold change ≥0.585, we identified 630 differentially expressed genes across treatment conditions (Figures 6C,D). Consistent with cell viability assays, the most pronounced transcriptional changes were observed with 10 µM regorafenib, which strongly downregulated genes involved in “cell cycle progression” and “cell division”, while upregulated genes related to “cell adhesion” and “cell motility” (Figure 6E). In contrast to epithelial-derived organoids, fibroblasts appeared resistant to autogramin-2 and showed limited transcriptional changes in response to 4 µM regorafenib. The combination treatment did not significantly alter gene expression compared to regorafenib alone, with the exception of a few genes. These included *Spp1/osteopontin/Opn,* involved in myofibroblast activation, *Slfn2* reported to regulate quiescence in hematopoietic stem cells, and *Angptl4,* which plays a role in fibroblast activation (Figure 6F) (Gao et al., 2024; Warsi et al., 2022; Saito et al., 2023).

## 4 Discussion

In the present study, we aimed to gain a better understanding of the mechanisms underlying regorafenib resistance, with the goal of identifying new strategies to enhance cancer cell sensitivity to this drug. Regorafenib, a multi-kinase inhibitor, is primarily known for targeting angiogenesis through inhibition of the VEGF signaling pathway (Arai et al., 2019). Here, we focused on investigating the drug’s effects on both normal and *Apc*-deficient epithelial cells. Using a model based on primary cultures of mouse colon organoids, we provide evidence that this reductionist, orthologous system predicts the induction of EMT, a known mechanism of chemoresistance, upon treatment with clinically relevant concentrations of regorafenib (4–8 µM). Importantly, this EMT response could be attenuated by co-treatment with an autophagy inhibitor.

Transcriptomic analysis of regorafenib-treated organoids identified both epithelial and tumor-derived cells as direct targets of the drug. Regorafenib exposure induced EMT, a well-established contributor to chemoresistance (Chen et al., 2022; Kehagias et al., 2022). Upon treatment, epithelial cells began expressing genes typically associated with the stromal compartment, including extracellular matrix components and signaling molecules. This cellular plasticity may render epithelial cells less dependent on signals from their surrounding microenvironment. Among the genes upregulated by regorafenib in organoids, *Inha* is of particular interest; it has been shown to be overexpressed in cancer-associated fibroblasts compared to normal fibroblasts and is implicated in tumor progression (Zhang et al., 2024; Li et al., 2023). Furthermore, proteins induced in epithelial cells by regorafenib may contribute to tumor immune modulation. For instance, *Thbs1*, commonly overexpressed in the colonic stromal compartment, is thought to promote immunosuppression in CRC, while *Spp1/Opn* marks macrophages involved in tumor immune evasion (Omatsu et al., 2023; Li et al., 2023). In addition, *Opg* has been implicated in the regulation of CD4^+^ T cell infiltration into the tumor microenvironment (Zhang et al., 2021). The observed downregulation of *IL18* following regorafenib treatment in organoids may further contribute to the development of a local immunosuppressive milieu (Li et al., 2021).

As regorafenib represents the last line of treatment for chemorefractory CRC patients, there is an urgent need to identify strategies to overcome regorafenib-induced resistance. In this study, we propose that targeting autophagy may offer a promising therapeutic approach. Autophagy is a physiological process that enables the recycling of cellular components and is involved in the cellular stress response to nutrient deprivation (Mulcahy Levy and Thorburn, 2020). In cancer, the role of autophagy is complex and context-dependent: it can act as a tumor suppressor during the early stages of cancer development, but under drug-induced stress, it may support tumor progression (Mahgoub et al., 2022). Consistent with the observed upregulation of phosphorylated Erk1/2 activity and increased autophagosome density in regorafenib-treated organoids, autophagic flux has been associated with the activation of this signaling pathway (Bishnu et al., 2021). In the present study, we provide transcriptomic, proteomic, and cellular evidence that combining autophagy inhibition with regorafenib treatment can help overcome drug-induced EMT and its associated secretory phenotype, leading to reduced cell proliferation and increased apoptosis. These findings initially observed in mouse organoids were confirmed in several human CRC cell lines, where combined treatment decreased the regorafenib IC50 by 2- to 5-fold. This reduction in the required drug concentration may help limit regorafenib-induced toxicity in normal adjacent tissues. Our results align with recent literature highlighting the complex interplay between autophagy and EMT in cancer, with EMT promoting autophagy and autophagy, in turn, regulating EMT either positively or negatively depending on the cellular context (Strippoli et al., 2024). Notably, although EMT and autophagy appear to play essential roles in regorafenib resistance, this does not exclude the involvement of other molecular mechanisms. In contrast, fibroblast cells (the main contributors to the stromal compartment) were much more resistant to regorafenib at the same concentrations. This suggests that the multi-kinase inhibitor preferentially targets epithelial cells in the colon, which exhibit a higher proliferative capacity compared to stromal fibroblasts.

Consistent with previous literature showing variability in autophagic flux among CRC cell lines, we found that HCT 116 cells were more sensitive to autophagy inhibition than SW480 or LoVo cells (Lauzier et al., 2019). Targeting autophagy in CRC has recently emerged as a promising therapeutic strategy (Ma et al., 2023). A recent phase I clinical trial evaluated the combination of the autophagy inhibitor hydroxychloroquine and a histone deacetylase (HDAC) inhibitor (entinostat) with regorafenib in metastatic CRC patients. However, this triple therapy was poorly tolerated and showed limited efficacy (Karasic et al., 2022). However, more potent and less toxic autophagy inhibitors, such as Lys05 and DC661, have recently been developed. Whether these compounds can suppress the regorafenib-induced profile as effectively as autogramin-2 remains to be determined in future experiments using our preclinical models.

A limitation of the current study lies inherently in the reductionist approach chosen, which focuses solely on investigating the impact of regorafenib on the epithelium. Recent publications have shed new light on the crosstalk among various cell populations within the tumor microenvironment, highlighting the instructive role that CRC tumor cells and stromal fibroblasts can play in modulating immunosuppressive states in immune cells (Li et al., 2023; Gao et al., 2024). Future co-culture experiments—using organoids and immune cells in the presence or absence of fibroblasts—should help elucidate the potential role of the regorafenib-induced organoid secretome in the crosstalk with colon stromal cells.

In summary, by employing a preclinical organoid model, our study highlights the potential of combining autophagy modulation with regorafenib to reduce treatment resistance in heavily pretreated mCRC patients. Future research should explore this therapeutic strategy using patient-derived tumor organoids (PDTOs) within a personalized medicine framework.

## Data Availability

The datasets presented in this study can be found in online repositories. The names of the repository/repositories and accession number(s) can be found in the article/Supplementary Material.
