# Supplementary material for: Autophagy inhibition improves sensitivity to the multi-kinase inhibitor regorafenib in preclinical mouse colon tumoroids
Source: Front Cell Dev Biol. 2025 Jul 23;13:1631116. doi: 10.3389/fcell.2025.1631116 (PMC12325355; doi:10.3389/fcell.2025.1631116)

Supplementary Material

Agostini et al.

1 Supplementary Data

Supplementary Material should be uploaded separately on submission. Please include any supplementary data, figures and/or tables.

Supplementary material is not typeset so please ensure that all information is clearly presented, the appropriate caption is included in the file and not in the manuscript, and that the style conforms to the rest of the article.

2 Supplementary Figures and Tables

For more information on Supplementary Material and for details on the different file types accepted, please see here.

Table 1

Table 1. List of cells used in this study

Organoid line	Mouse genotype	Age	Sex
F4 (2)	VilCreERT2 Heterozygous/Apc Wild-type	2 months old	Female
M1	VilCreERT2 Heterozygous/Apc Wild-type	2 months old	Male
M40	VilCreERT2 Heterozygous/Apc Wild-type	2 months old	Male
M41	VilCreERT2 Heterozygous/Apc Wild-type	2 months old	Male
F10	VilCreERT2 Heterozygous/Apc Δ	2 months old	Female
F11	VilCreERT2 Heterozygous/Apc Δ	2 months old	Female
M3	VilCreERT2 Heterozygous/Apc Δ	2 months old	Male
M14	VilCreERT2 Heterozygous/Apc Δ	2 months old	Male

CRC cell line	CMS subtype	Age	Gender	Organ
LoVo	CMS1	56	Male	Large intestine; Colon
DLD-1	CMS1	/	Male	Large intestine; Colon
HT29	CMS3	44	Female	Large intestine; Colon
HCT116	CMS4	/	Male	Large intestine; Colon
SW480	CMS4	50	Male	Large intestine; Colon

Fibroblast line	Mouse genotype	Age	Sex
F1	Wild-type	2 months old	Female
F3	Wild-type	2 months old	Female
F4(2)	Wild-type	2 months old	Female
M1	Wild-type	2 months old	Male
M2	Wild-type	2 months old	Male
M3	Wild-type	2 months old	Male
M3(2)	Wild-type	2 months old	Male

Table 2

Table 2. List of products used in this study

**Compound Name**	**Supplier**	**Cat. No.**
Regorafenib	Selleckchem.com	S1178
Autogramin-2	MedChemExpress	HY128340
**Cell culture reagents**	**Supplier**	**Catalog number**
**Organoids**		
Advanced-DMEM/F12 medium	Thermo fisher scientific	12634028
DMEM	Gibco	41965-039
Wnt3a-conditioned medium	ATCC	CRL2647
L-Glutamax	Thermo fisher scientific	35050061
N-2 Supplement	Thermo fischer scientific	17502048
B27 Supplement w/o vitamine A	Thermo fisher scientific	12587010
Amphotericin	Thermo fisher scientific	15290026
Gentamycin	Thermo fisher scientific	15750037
Penicillin-Streptomycin cocktail	Thermo fisher scientific	15070063
UltraPure EDTA	Invitrogen	15575038
HEPES 1M	Thermo fisher scientific	15630080
N-Acetyl-L-cysteine	Sigma Aldrich	A9165
Recombinant Murine Noggin	PeproTech	250-38-250UG
Recombinant Murine R-Spodin-1	PeproTech	120-38-100UG
Recombinant Murine EGF	PeproTech	315-09-500UG
Nicotinamide	Sigma Aldrich	N0636
TryplExpress	Thermo fisher scientific	12605028
Matrigel Matrix Basement Membrane	Corning	354234
Y-27632 dihydrochloride (ROCK inhibitor)	Sigma Aldrich	Y0503
100 µm cell strainer	VWR	10054458
70 µm cell strainer	Corning	352350
Sunflower seed oil	Sigma	S5007
Ethanol absolute	VWR	20821-296
Fetal bovine serum (FBS)	ThermoFisher	10270106
DPBS	Thermo fisher scientific	14190094
**Fibroblast lines**		
DMEM/F-12	Thermo fisher scientific	11320033
Collagenase D	Sigma Aldrich	11088882001
Dispase	Gibco	17105-041
**CRC lines**		
EMEM with L-Glutamine	ATCC	80603245
McCoy'S 5A w/L-Glutamine	VWR	392-0420
RPMI Medium 1640	Gibco	A10491-01
Trypsin 2.5%	Gibco	15090-046
Crystal Violet	Sigma Aldrich	C0775-25G
Formalin solution	Sigma Aldrich	HT5011
Poly-L-Lysine solution	Sigma	P4707
Triton X100	Sigma Aldrich	T8787
**Immunohistochemistry/ immunofluorescence**	**Supplier**	**Catalog number**
Bovine serum albumin	Sigma Aldrich	A3294
Coverquick 4000	VWR Chemicals	5547539
Fluorsave reagent	Millipore	345789
Horse serum	ThermoFisher	16050122
Mayers' hemalun solution	Millipore	1092492500
Sucrose	Millipore	1076511000
Sodium citrate	VWR	27833,294
Tissue freezing medium	Leica	14020108926
Alexa Fluor® 488 AffiniPure® Donkey Anti-Rab	Jackson ImmunoResearch	711-545-152
DAPI	Sigma-Aldrich	D9542
Rabbit anti-BrdU	Abcam	AB6326-250
Rabbit anti-LC3B (D11)	Cell Signaling	3868
Rabbit p-42/44 (T202/Y204) (MAPK)	Cell Signaling	9101
TUNEL assay	Merck	11684795910
Mouse Osteoprotegerin/ TNFRSF11B Antibody	Biotechne	AF459
Mouse Osteopontin/ OPN Antibody	R&D systems	AF808
**RNA extraction**		
RNeasy Mini Kit	Qiagen	74106
**RNAscope probes**	**Supplier**	**Catalog number**
Mouse Axin2	Biotechne	2400331
Mouse Bmp1	Biotechne	311151
Mouse Il18	Biotechne	416731
Mouse Inha	Biotechne	485661
Mouse Thbs1	Biotechne	457891
**Proteomic studies**	**Supplier**	**Catalog number**
Pierce™ BCA Protein Assay Kits	ThermoFisher	23227
Cell Recovery Solution	Corning	354253
p44/42 MAPK (Erk1/2) Antibody	Cell Signaling Technology	9102
Goat anti-Rabbit IgG, Secondary Antibody, HRP	Invitrogen	31460
Tween	BioRad	1706531
Chemiluminescent Substrate	ThermoFisher	34580
Mouse XL Cytokine Array	R&D Systems	ARY028
Proteome profiler phospho-kinase array kit	R&D Systems	ARY003C
Protease Inhibitor Cocktail	Roche	11836145001
**Oligonucleotides for qRT-PCR**	**Forward primer**	**Reverse primer**
Mouse Ascl2	AAGCACACCTTGACTGGTACG	AAGTGGACGTTTGCACCTTCA
Mouse Axin2	TGACTCTCCTTCCAGATCCCA	TGCCCACATAGGCTGACA
Mouse Gapdh	TGCACCACCAACTGCTTAG	GATGCAGGGGATGATGTTC
Mouse Gpc1	AGTGTCATTGGCGGTGTGC	GGATTGACCTTGGGGTTTCCA
Mouse Mex3a	TTCTTTCCATCAGCCCCAGC	TGTCAATCACTGGTGTCCCG
Mouse Ptk7	TTGAACCTCGCTGCTCGTCT	GACGATAGCGGCTTGGGC
Mouse Thbs1	CCCTGGACTTGCTGTAGGTTA	GCTGGACTGGTAGCCGAAAA
Mouse Tnfrs11b	GCCACGCAAAAGTGTGGAAT	TCCACCAAAACACTCAGCCA
Mouse Yap	TATTTCGGCAGGAATTAGCTCT	TGAGACATCCCAGGAGAAGACA
Mouse Ywhaz	TGCAACGATCTACTGTCTCTT	CGGTAGTAGTCACCCTTCATTTTCA
**Softwares**		
GraphPad Prism 10	https://www.graphpad.com	
qPCR 4.0	Analytik Jena	
qBase	Biogazelle	
GSEA MolSig	Broad Institute	
Degust	Monash Institute	
ZEN Blue 3.5	Zeiss	
NDP.view2	Hamamatsu	
Affinity Designer 2		
Image J		
**Equipment**	**Microscope-mounted camera**	
BIO RAD iMark Microplate Reader		
Cryostat Leica Microsystems GmbH		
Microscope Zeiss Axio Observer 3/5/7 KMAT	Axiocam 503 mono	
Microscope Moticam Pro	Motic AE31	
NanoZoomer S360		
qTower Touch 96 Analytik Jena		

2.1 Supplementary Figures

frontiers

Supplementary Figure 1.

A. Gene expression analysis by qRT-PCR of the indicated genes following culture of organoids under the indicated conditions. Each symbol corresponds to a given tumoroid line. Expression levels are normalized to that of reference genes (*Gapdh* and *Ywhaz*). No statistics applied (n=2).

B. Expression levels of genes differentially modulated in Apc-deficient vs Apc wild-type organoids in Vehicle (Ve) conditions. CP20M: counts per kilobase of transcript per 20 million mapped reads. Unpaired t test with Welch's corrections.

C. Expression levels of genes coding for EMT- and stem cell-related markers. CP20M: counts per kilobase of transcript per 20 million mapped reads. Vehicle (Ve), 4 µM regorafenib (R4) or 8 µM regorafenib (R8). Each symbol corresponds to the value of an organoid line generated from an individual mouse. One way ANOVA test with Tukey's multiple comparisons.

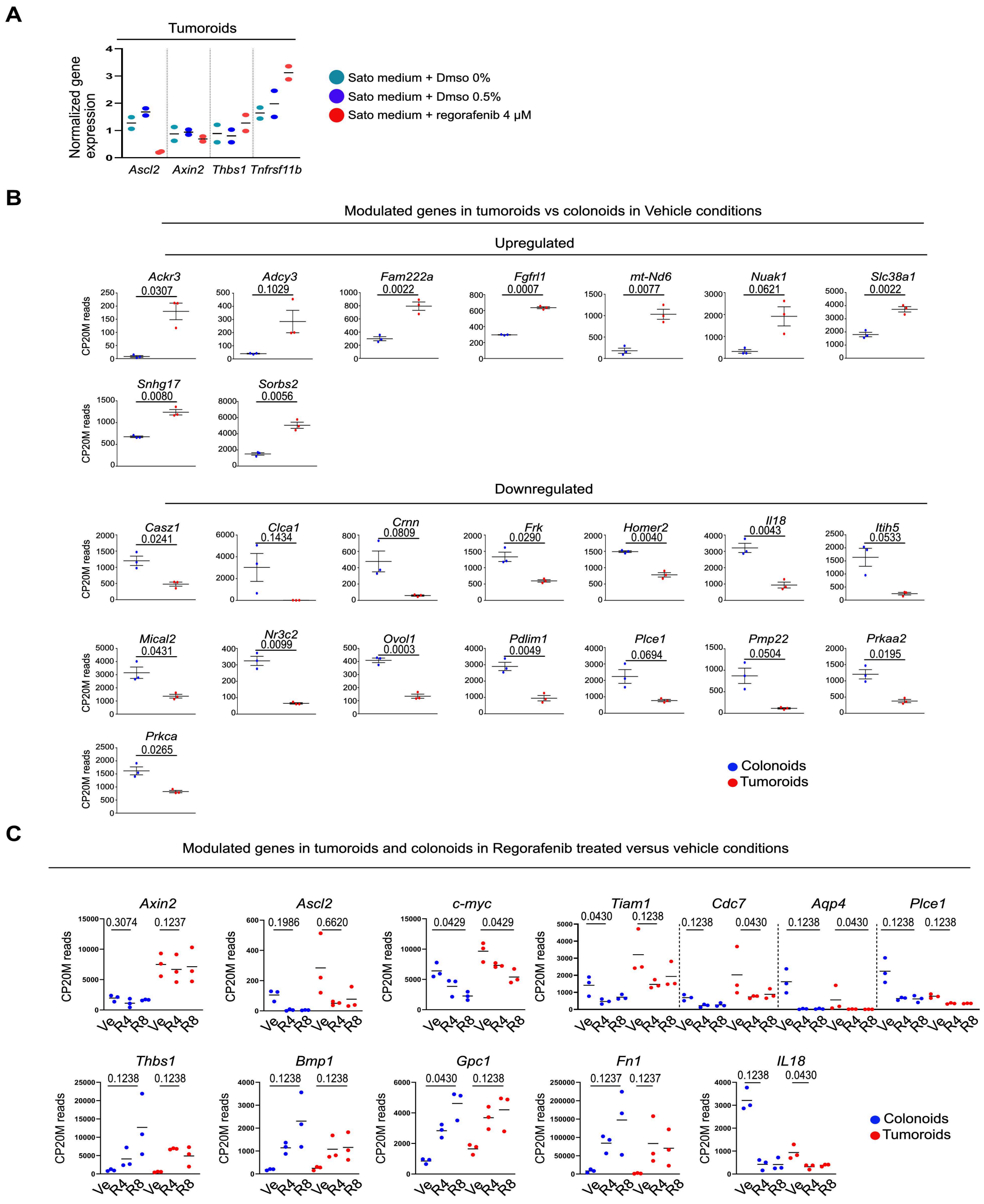

Supplementary Figure 2.

A. Representative pictures of immunofluorescence showing expression of LC3B in vehicle or drug-treated tumoroids. Nuclei counterstained with Dapi. Scale bars: 50 µm.
B. Quantification of LC3B^{+ve} punctuates/cell to visualize autophagosomes. For each organoid line, data were expressed relative to vehicle (Ve) conditions (set to 1.00). A mean of 273 cells were analyzed per organoid line per condition. One-way ANOVA tests with Tukey's multiple comparisons. Ve vs R4: p=0.0637; Ve vs A1: p=0.2901; Ve vs R4A1: p=0.0274; R4 vs A1: p=0.987; R4 vs R4A1: p=0.0417; A1 vs R4A1: p=0.0221.

A

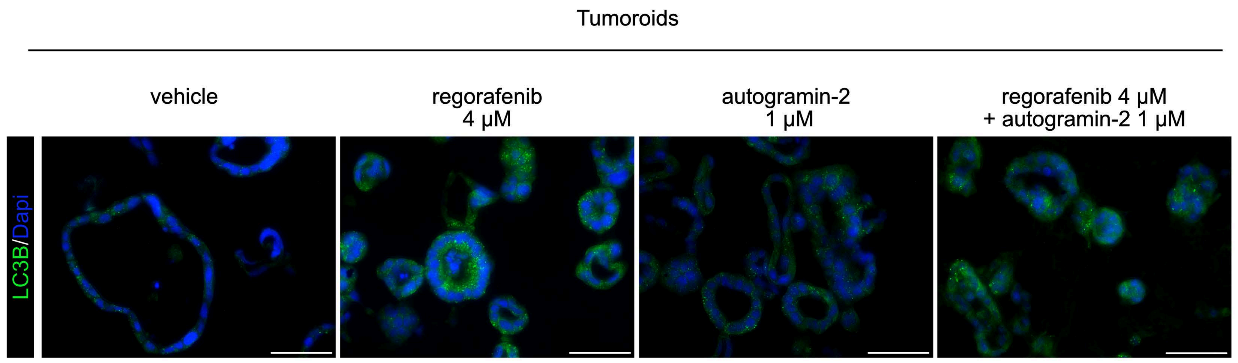

B

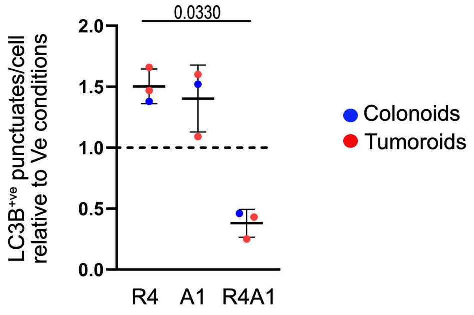

Supplementary Figure 3.

Expression levels of genes coding for colon fibroblast subtype markers. CP20M: counts per kilobase of transcript per 20 million mapped reads. Each symbol corresponds to the value of an organoid line generated from an individual mouse.

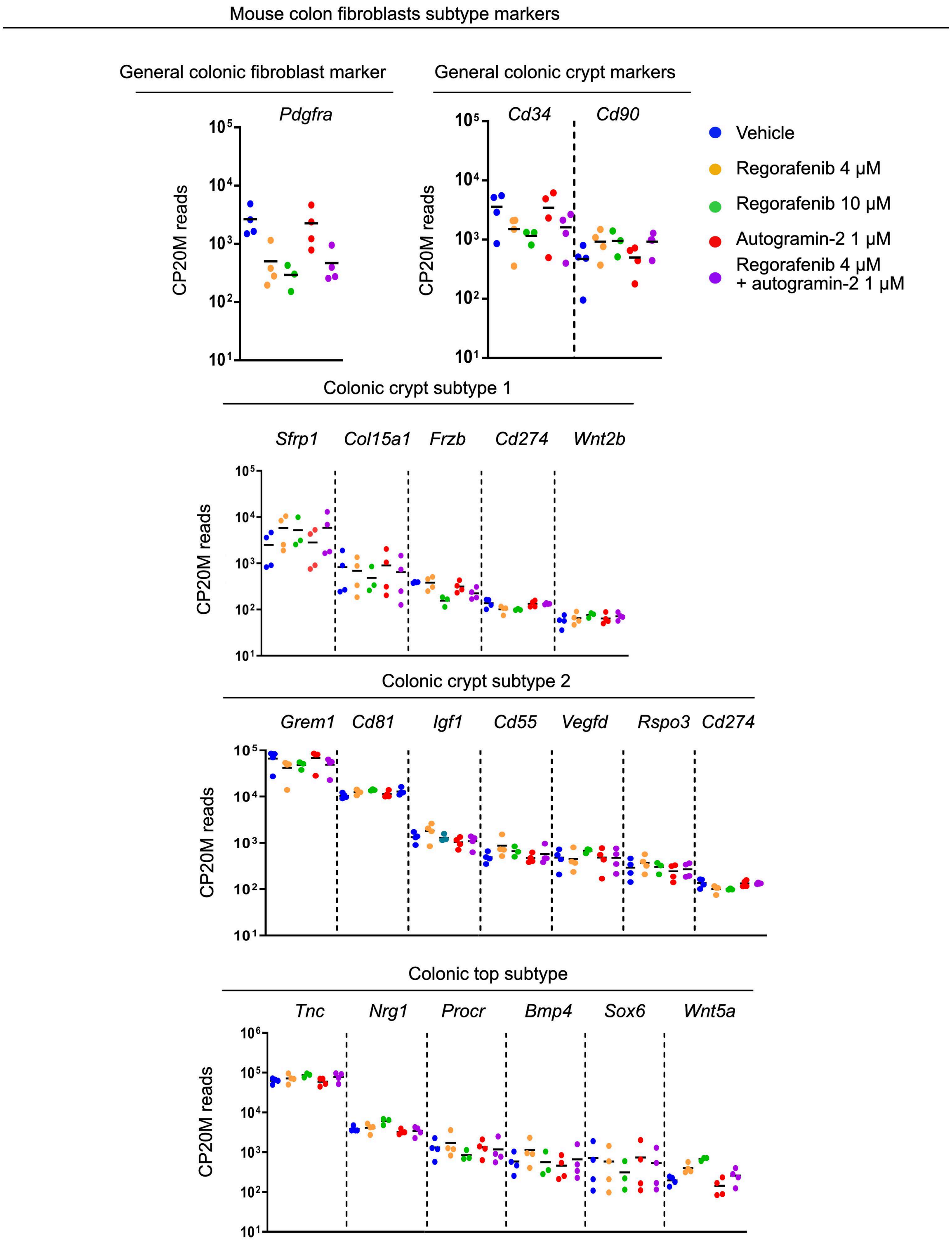

Supplement: Supplementary file 1 [file DataSheet1.pdf]
